# Work Support, Role Stress, and Life Satisfaction among Chinese Social Workers: The Mediation Role of Work-Family Conflict

**DOI:** 10.3390/ijerph17238881

**Published:** 2020-11-29

**Authors:** Cindy Xinshan Jia, Chau-kiu Cheung, Chengzhe Fu

**Affiliations:** 1 Department of Social Work, College of Public Management, South China Agricultural University, Guangzhou 510640, China; cindyjia@scau.edu.cn; 2 Department of Social and Behavioural Sciences, City University of Hong Kong, Hong Kong, China; c.j@cityu.edu.hk; 3 School of Politics and Public Administration, South China Normal University, Guangzhou 510631, China

**Keywords:** life satisfaction, role stress, social workers, work-family conflict, work support

## Abstract

The current study examined the relationships among work support, role stress, work-family conflict, and life satisfaction, with a sample of social workers in China’s Pearl River Delta (*N* = 1414). Using structure equation modelling, the study revealed that social workers’ life satisfaction reduced because of role conflict and work-family conflicts. Work-family conflict partially mediated the negative effects of role ambiguity and conflict on social workers’ life satisfaction. Work support from their director, manager, supervisor, and co-workers protectively reduced role stress and work-family conflict. The findings emphasize the significance of managing the interference between work and family for social workers’ well-being.

## 1. Introduction

Social workers’ well-being has attracted growing attention due to the contamination of their work experiences [1,2,3]. Providing emotional labor, social workers are one of the at-risk professionals in experiencing negative work-family interference with exposures of various negative work events [4,5,6,7]. Workplace experiences, such as stress and burnout [8,9,10,11,12], are likely to extend into the domain of their private life and to lead to high risks of negative work-to-family spill-over that could impair their life satisfaction [9]. While the social work literature has mainly focused on the negative pathways from social workers’ stress and strains to their problematic work outcomes [13], research on the influence of work-family conflict (WFC) on social workers’ life satisfaction remains sparse in the social work context (see exceptions of [9,13,14,15]).

Like the global situation, social workers in China also confront risks to their quality of life, which has been scarcely studied. With the rapid development of social work in Mainland China in the past three decades, social workers face high workplace stress [16], experience high turnover [17], and low job satisfaction [18]. Besides the common risk factors (see a meta-analysis of [19]), the recent transitions occurring in community-level social work organizations in China’s Pearl River Delta (PRD) are creating work stress. As government procurement in social services led the nationwide development in Chinese social work starting from 2006 in PRD, Shenzhen, a major PRD city, has initiated a community-level administrative transition in the past four years. Social work services are integrating with community councils (the primary administrative unit of governance). These integrated service centers require community social workers to cooperate with local administrators in managing community affairs. As such, social workers are now required to meet the demands of social work service on one side, and political governance on the other [20], which possibly increase their role stress. 

Moreover, the negative work-family spill-over onto one’s personal life might be more pronounced in a Chinese cultural context. The separation between work and non-work domains in Chinese cultural tend to be an unclear [21,22]. As a collectivism culture [23], the Chinese culture views work-family negative spill-over as an inevitable cost to promote financial status for the family [24]. Compare with the United States, the association between work demands and WFC was more pronounced in China [25]. As such, negative work experiences may severely impact the life satisfaction of Chinese social workers. 

Nevertheless, social workers’ life satisfaction remains largely under-examined, specifically with regards to the work-family negative spill-over in the context of administrative transitions. Thus, the current study aims to examine the structural pathways linking work support, role stress at workplace, and WFC to life satisfaction with a representative sample of social workers in the PRD of China.

## 2. Literature Review

### 2.1. Role Stress, Work-Family Conflict, and Life Satisfaction

As “a cognitive assessment of satisfaction with life circumstances” [26], life satisfaction (LS) serves as an important aspect of quality of life regarding the occupational health [27,28]. In social work context, LS is influenced by various predictors, such as one’s professional self; work commitments, autonomy, overload, and types of work; organizational culture and environment; professional principles; work-family interference; work satisfaction; and social interactions at work [5,6,9,29,30,31]. Among these factors, the work-family interference on LS was limited explored in the social work context.

Work-family interference rests on the spill-over hypothesis, which posits that individuals’ working experiences spill over to other life domains and influence LS positively or negatively [22,32,33,34]. The negative spill-over between work and family, called work-family conflict, refers to “a form of inter-role conflict in which the role pressures from the work and family domains are mutually incompatible…” [35] (p. 77). WFC occurs the demands of work or family role hinder it to fulfil the demands in the other role [35]. Thus, WFC includes two bidirectional influences: work’s interference on the family (WIF) and family’s interference on work (FIW) [36,37]. 

WFC possibly mediates between stress and LS. Role stress, as a type of work stress caused by uncertainties about one’s job duties, work demands, and overload [38], leads to WFC [39,40,41]. Subsequently, WFC leads to negative work outcomes (e.g., burnout, turnover, and job dissatisfaction), and reduced LS were generally found [36,42,43,44]. Moreover, the mediation effect of WFC between stress and LS was directly evidenced among the general population [45,46].

Social workers are particularly vulnerable to WFC as their professional work usually extends into personal life [2]. They are confronted by the negative effects of therapeutic work [47], empathy burnout [48], compassion fatigue, and vicarious trauma from their clients’ experiences [11,49], as well as the risks of actual violence [50,51]. As such, social workers are exposed to role stress of ambiguity and conflict [52,53] and experienced WFC [15]. In addition, WFC tends to be detrimental to the Chinese [25,54,55]. Compared with individualistic cultures, Chinese culture tend to emphasize work as a supportive way to family and an inevitable cost to family financial stability [24,56].

Nevertheless, studies exploring the influence of role stress and WFC on social workers’ LS remain sparse. Regarding a few studies about WFC’s negative influences among social workers, gender and job hierarchical differences in WFC have presented [14], and WFC’s negative influences on work satisfaction have been evident [13,15]. Regarding the quality of life, WFC’s negative effect on general well-being was found among child welfare workers [57]. Another qualitative study noted that social workers who achieve a work-life balance show improvement in subjective well-being [9]. In a Chinese context, only one study employed a sample of 829 social workers that, Wang and colleagues examined the influence of job autonomy, work-family enrichment (WFE) and job satisfaction on turnover intention [17]. Their results suggested WFE significantly influence job satisfaction but failed to support WFE mediating between job autonomy and turnover intention. Therefore, the mechanism of role stress, WFC, and LS needs further clarification.

Based on previous studies reviewed, we hypothesize WFC mediates between role stress and LS among social workers:

**Hypothesis** **1 (H1).** *That role stress (i.e., role ambiguity, role conflict, and role overload) positively predicts WFC (i.e., WIF and FIW) and negatively predicts LS.*

**Hypothesis** **2 (H2).** *That WFC negatively predicts LS.*

**Hypothesis** **3 (H3).** *That WFC mediates between role stress and LS.*

### 2.2. The Protective Role of Work Support

Work support, is defined as psychological or material resources provided through social relationships that can mitigate work stress or strain at workplace [58]. Based on the main effect hypothesis of social support on stress [59,60], social support serves to manage stress, mitigate strain, and maintain well-being. That is, supportive relationships protect one from stress or strain, and thus enhance one’s LS [61]. The literature demonstrated that work support eases work stress [39,62,63] and WFC [64,65]. For example, work support by supervisors and coworkers helps to reduce the role stress caused by overload, and leads to less WFC [66]. Further, meta-analyses have revealed that social support directly and indirectly improves one’s LS [58,67]. 

In the social work domain, there has been a lack of clarity on how the mechanism of social support influence WFC and LS. By mitigating social workers’ stress or strain [11,29], social support protects social workers from negative work experiences, such as burnout [5,68] and promotes subjective well-being [9]. Of the few studies that considered WFC, one revealed that social workers took advantage of work support to cope with WFC [15]. Lizano and Mor Barak [69] revealed that work support moderated the influence of role stress and work-family conflict on burnout and job satisfaction among social welfare workers. Kalliath and colleagues found that at a higher level of family support, WFE promoted Indian social workers’ work well-being and satisfaction [70]. In the Chinese context, possible cultural differences of the perceived availability of social support [71] or appropriateness of seeking social support [72] might appear. However, how social support protects the impact of WFC and role stress on LS were largely uncharted, especially in the Chinese social work context. Based on previous reviewed studies, we further propose the following hypotheses:

**Hypothesis** **4 (H4).** *Work support from one’s director, direct manager, supervisor, and co-workers, negatively predicts role stress and WFC, and positively predicts LS.*

## 3. Materials and Methods 

### 3.1. Data and Sample

The current study employed a subsample from the Pearl River Delta from the first-wave data from the Chinese Social Work Longitudinal Survey conducted in 2019 (CSWLS 2019) [73,74]. CSWLS 2019 is the first national-wide survey of social work organizations and social workers. The ethical approval for CSWLS 2019 was achieved by the research ethics committee in a Chinese university [74]. The survey employed paper-pencil questionnaires with a stratified random sampling method, and surveyed social work agencies and social workers from 56 cities of 22 provinces in Mainland China [73,74]. First, a selected pool of social work organizations formed according to the Chinese Social Organization Platform. Then, 33 social work agencies were randomly selected from the capital city or municipality, while 11 social work agencies per city were randomly selected for the rest. At last, 10–30 social workers were randomly selected in every agency according to the agencies’ total number of employees [74]. Informed consent was obtained [73,74].

The current study employed its PRD subsample of CSWLS2019, which including nine cities of Dongguan (DG), Foshan (FS), Guangzhou (GZ), Huizhou (HZ), Jiangmen (JM), Shenzhen (SZ), Zhaoqing (ZQ), Zhongshan (ZS), and Zhuhai (ZH) in Guangdong Province. This subsample is a representative microcosm of social work development in Mainland China. PRD is a leading area in Mainland China for social work development, as reflected in its highest number of social work agencies and qualified social workers [75]. Moreover, the organizational transition of combining social work service with community councils was widely adopted by several PRD cities such as Guangzhou and Jiangmen. 

### 3.2. Measures

The current study employed social worker dataset in PRD from CSWLS 2019 and includes the following measures:

#### 3.2.1. Outcome Variable

Life satisfaction was measured by the single-item global scale [76] with “how satisfied are you with your life as a whole?” in C16 section of the questionnaire. It was originally measured by a 5-point rating scale from “Very Satisfied” = 1 to “Very Unsatisfied” = 5. We transformed to a reverse-coding according to the global scale [76]. A higher score presents a greater degree of LS.

#### 3.2.2. Independent Variable

Work support was measured by the validated Chinese version of House and Wells’s scale (I section) [77,78]. Four types of work support sources were asked about, including agency director, direct manager, social workers’ supervisor, and co-workers. It was measured on a 5-point scale from “Very Unsatisfied” = 1 to “Very Satisfied” = 5. A composite of these four sources had higher scores representing greater perceived work support. The reliability (Cronbach’s α) ranged from 0.89 to 0.92.

#### 3.2.3. Mediator

Role stress was measured by role ambiguity and conflict, as well as role overload. The validated Chinese version of 6-item role ambiguity and 8-item conflict scale [79,80] were used to measure role stress (H section). The responses ranged on a 5-point Likert scale of agreement from “Strongly Disagree” = 0 to “Strongly Agree” = 4. The role ambiguity and conflict subscales yielded good reliability (Cronbach’s α = 0.73 and 0.76, respectively). Followed Rapoport and Rapoport [81], role overload was indicated by time-based work overload with the question “In general, how much overtime work do you do?” (D28D). The response of “None” was coded as “0,” whereas all other options of working overtime on workdays, weekends and holidays were coded a “1.”

Work-family conflict was measured by the validated Chinese version of Work-Family Balance Scale in S section of the survey [82,83]. The responses ranged on a 5-point agreement scale from “Strongly Disagree” = 0 to “Strongly Agree” = 4. Two subscales of 3-item WIF and 4-item FIW yielded good reliability (Cronbach’s α = 0.82 and 0.86). The composed scores of WIF and FIW were used, and higher scores represented greater conflicts in work-to-family interference and family-to-work interference, respectively.

#### 3.2.4. Controlled Covariates

Job tenure refers to years of working as a social worker. As a longer tenure presents accumulated resources in the workplace [84], it was controlled. We transformed it from the date format (D3A) into years of tenure.

Social work qualifications (B7) was a control factor because such professional qualifications may lead to differences in work contents or coping strategies [15]. The three levels of social work qualifications in Mainland China were: Assistant, junior, and senior level. As the senior qualification began in 2019, only two levels existed in the dataset. The qualifications of assistant and junior social workers were dummy-coded, respectively. 

#### 3.2.5. Demographic Characteristics 

As marital status, gender, age, and years of education influence WFC and LS, these characteristics are used for control purposes [3,39]. As divorced or widowed cases were limited, marital status (A9) was dummy-coded into “married” = 1, and “single/divorced/widowed” = 0. Gender (A1) was coded as “female” = 1 and “male” = 0. Age (A2) was transferred from birth date to years of age. Education (B1) was recoded into years of education for 9-19 years. 

### 3.3. Data analysis

**Figure 1:**
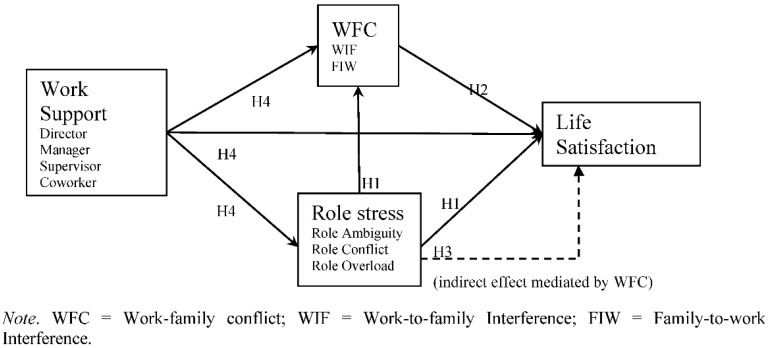
Theoretical model and research hypotheses of work support, stress, WFC, and life satisfaction (LS).

Regarding missing data, as CSWLS 2019 employed return visits to re-fulfil the data, the proportions of missing data were smaller than 4.60% among all variables and thus the following analysis was performed by default. Zero-order correlations and structure equation modelling (SEM) with maximum likelihood (ML) estimation were conducted with Stata 15 (StataCorp LLC, College Station, TX, USA) for hypotheses testing. A mediation model with multiple mediators was properly tested with SEM [85]. The indirect effect of mediation was tested through the Monte Carlo method, as it was preferred for SEM [86]. As the current study aimed to understand the protective role of general work support than the specification of sources, work support from the director, manager, supervisor, and co-workers was modelled as a latent variable to capture the shared variance of work support [87]. According to the hypotheses (Figure 1), work support was estimated to predict role stress (i.e., role ambiguity, role conflict, and role overload), WFC (i.e., WIF and FIW), and LS. Role stress was estimated to mediate between work support and WFC, as well as between work support and LS. WFC was estimated to mediate between work support and LS, as well as between stress and LS. Controlled variables were estimated as covariates for independent, mediating, and outcome variables. 

## 4. Results

The demographic characteristics of the PRD subsample in CSWLS 2019 are presented in Table 1. Although the response rate was not available according to the survey report [73], the average age of the PRD subsample was 29.21 (see Table 1). This statistic was highly close to the average age of 29.37 reported by the Social Work Association of Shenzhen city [73], which partially supported the sample representativeness.

The intercorrelations between independent variables, mediators, and outcome variables are presented in Table 2. According to Akoglu [88], as expected, the four sources of work support were moderately inter-correlated (i.e., *r* = 0.55~0.58, *p* < 0.00). Work support exhibited weak and negative correlations with role ambiguity (RA) and conflict (RC) (*r* = −0.29~−0.36, *p* < 0.00), whereas work support (except from managers) was slightly negatively correlated with role overload (RO) with *r_pb_* (the Point-biserial *r*) = −0.06~−0.08, *p* < 0.05. The correlation coefficients between work support and WFC were negative, as expected, though small (*r* = −0.13~−0.20, *p* < 0.00). RA and RC were moderately positively inter-correlated (*r* = 0.43, *p* < 0.00), and slightly correlated with RO (*r_pb_* = 0.09~0.10, *p* < 0.01). WIF and FIW were moderately inter-correlated (*r* = 0.54, *p* < 0.00). The correlation coefficients between WIF, FIW, and three types of role stress were positive as expected (*r/ r_pb_* = 0.11~0.35, *p* < 0.00), though weak. LS was slightly positively correlated with work support (*r* = 0.08~0.10, *p* < 0.01; except the director’s support), slightly negatively correlated with role stress (*r/r_pb_* = −0.09~−0.20, *p* < 0.00), WIF and FIW (*r* = −0.20~−0.22, *p* < 0.00). 

**Figure 2:**
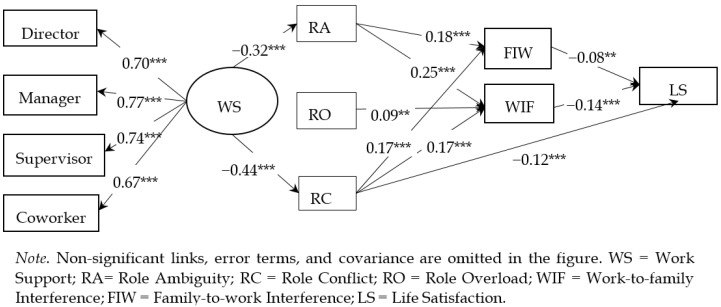
The standardized structure equation modelling (SEM) results of work support, role stress, WFC, and LS.

The SEM results and model-fit index are illustrated by Figure 2 and Table 3. In general, the model yielded a good model fit according to Hu and Bentler [89]: $\chi^{2}/df$ = 2.62 < 3, the Comparative Fit Index (CFI) = 0.98 > 0.90, the Tucker-Lewis Index (TLI) = 0.94 > 0.90, the Root Mean Square Error of Approximation (RMSEA) = 0.04 < 0.05. H1 was partially supported. RA and RC positively predicted both WIF (*β* = 0.25, *p* < 0.00; *β* = 0.18, *p* < 0.00) and FIW (*β* = 0.17, *p* < 0.00; *β* = 0.17, *p* < 0.00), whereas RO only positively predicted WIF (*β* = 0.09, *p* < 0.01). Only RC negatively predicted LS (*β* = −0.12, *p* < 0.00), net of other effects. H2 was supported that, WIF (*β* = −0.14, *p* < 0.00) and FIW (*β* = −0.08, *p* < 0.00) both negatively predicted LS. Regarding H3, the Monte Carlo method (5000 replications) showed that the indirect effect of FIW mediating between RA and LS was −0.01 (95% CI = −0.03~−0.00, *p* < 0.05), and between RC and LS was −0.03 (95% CI = −0.05~−0.02, *p* < 0.00). No significant effect of FIW mediating between RO and LS appeared. The indirect effect of WIF mediating between RA and LS was −0.03 (95% CI = −0.05~−0.02, *p* < 0.00), between RC and LS was −0.02 (95% CI = −0.04~−0.02, *p* < 0.00), and between RO and LS was −0.01 (95% CI = −0.02~−0.00, *p* < 0.05). H3 was thus supported except for the mediation effect of FIW between RO and LS.

Regarding H4, the latent variable of work support negatively predicted RA (*β* = −0.32, *p* < 0.00) and RC (*β* = −0.44, *p* < 0.00), while no significant predictive effect of work support for RO, WFC, or LS appeared. In addition, indirect effects of mediators between work support and LS were tested using the Monte Carlo method (5000 replications). Significant results appeared: The indirect effects of RA and RC mediating between work support and FIW were −0.06 (95% CI = −0.08~−0.04, *p* < 0.00) and −0.08 (95% CI = −0.11~−0.05, *p* < 0.00), respectively; the indirect effects of RA and RC mediating between work support and WIF were −0.08 (95% CI = −0.08~−0.06, *p* < 0.00) and −0.07 (95% CI = −0.10~−0.05, *p* < 0.00), respectively; the indirect effect of RC mediating between work support and LS was 0.05 (95% CI = 0.02~0.09, *p* < 0.00). 

Regarding demographic variables, it appeared that, the female social workers showed higher level of WIF (*β* = 0.06, *p* < 0.05) and LS (*β* = 0.05, *p* < 0.05), the elder ones were likely to experience less RO (*β* = −0.10, *p* < 0.05), the married ones were likely to experience less RA (*β* = −0.07, *p* < 0.05) and RC (*β* = −0.10, *p* < 0.01) but greater LS (*β* = 0.11, *p* < 0.01), and those with higher education were likely to perceive less WS (*β* = −0.09, *p* < 0.01).

## 5. Discussion

The current study examined the pathways among work support, role stress, WFC, and LS, with a representative sample of social workers from nine cities in PRD, China. To our knowledge, this is the first study to preliminarily explore social workers’ LS in relation to social support, WFC and role stress with quantitative method, especially in a Chinese context. It revealed that Chinese social workers’ LS reduced directly because of role conflict and WFC (i.e., WIF and FIW). Further, role ambiguity and conflict reduced LS indirectly through the mediation of WFC. Work support, as a protective factor, directly reduced role ambiguity, role conflict, and WFC. Moreover, work support indirectly benefited LS through lessening the negative influence of role ambiguity and conflict.

Work-family interference is noteworthy regarding social workers’ quality of life, especially among female workers. Coinciding with Yucel and Minnotte [46], we indicated that WFC directly impacted LS and aggravated the negative influence of role stress on LS. It contributes and adds to the literature of negative work-family spill-over perspective [35], by linking social workers’ WFC, role stress, and life quality [13,90]. Regarding the individualism–collectivism spectrum, compared with studies conducted in an individualism cultural setting [43], the current study appears not evident that WFC impairs LS to a greater extent [24]. Gender difference in WFC is worth noted that, we indicated that female social workers experience significantly greater WFC than did males (see Table 3), which was also in line with Baum’s findings [14]. 

Second, social workers’ role stress of role ambiguity and conflict at workplace were highlighted. The current study elaborates on how work stress worsened WFC and adds to the occupational health literature of social workers, especially in a Chinese cultural context [17]. As role stress is commonly reported among social workers [65,91], we highlighted that, role ambiguity influences WFC more than other variables, whereas role conflict was the only role stress factor that facilitated WFC and spontaneously reduced one’s LS, which echoes Michel and colleagues’ results of role conflict as the factor most associated with WFC [65]. It might result from the previous mentioned community-level administrative transition at PRD, which needs further exploration. In addition, we indicated as age increased, social workers face significantly less role overload, which possibly because of experience or skills (e.g., resilience) accumulated [92]. Furthermore, we found that married status reduced role ambiguity and conflict, which possibly stems from family support in promoting job involvement [93]. 

Third, the protective role of work support regarding social workers’ LS is evident. Work support from the director, direct manager, supervisor, and co-workers directly protects social workers from role stress, and mildly while indirectly promotes LS. Echoing the main effect hypothesis of social support on stress [59], we indicated that Chinese social workers employ a certain degree of resources derived from workplace social interactions in coping with role stress, which is also in line with findings in other cultural contexts [15]. Additionally, our result suggests that social workers with less education received more work support. A possible reason is that job mentoring is likely to happen among those with less professional education.

In addition, the current study indicates that social work job qualifications matter. Compared with those with no qualifications, assistant social workers faced greater role overload and WFC (Table 3). However, this effect did not present among junior social workers. Possible explanations of work tenure [14,94] and professional skills [95,96] are worth further research.

The limitations of this study are noteworthy. First, we only focused on workplace antecedents, and did not distinguish between the types of WFC [13]. Future study may expand to look at stress and support in the family domain and elaborate WFC. Moreover, as the current study rested on the main hypothesis of social support on stress, future research may elaborate the buffering hypothesis about the effect of social support on stress [57], and the sources or alternative protective mechanisms of work support [58]. Second, the dataset of CSWLS 2019 rests on a cross-sectional design of its first wave. Causal sequences need further exploration. For example, WFC was alternatively defined as a work-related stressor rather than a strain [35]. Longitudinal data, including those from the second or the third wave, are imperative for future work. Third, further cross-cultural comparisons are worthy of research in future. 

## 6. Conclusions

The current study highlights the negative impact of role conflict and WFC in reducing Chinese social workers’ LS. In addition, work support from the agency director, direct manager, supervisor, and co-workers protects social workers’ LS by lessening role conflicts. Social workers’ length of work, professional qualification, gender, and marital status are significantly associated with role conflict, WFC, and LS. It has several practical implications. First, the administrative practice of work support would be worth pursuing. As work support’s protective role in reducing social workers’ work stress is evident by the current study and others [59], administrative encouragement of social interactive collaboration among the workplace would optimize social workers’ performance. Second, possible administrative support handling work-family interference among female social workers would be recommended. As female social workers face greater WFC than do males in the current sample and general population [97,98], possible policy regarding parenting support or alternative work schedules is worth considering to improve female social workers’ work-family balance and well-being. Finally, interventions into social workers’ work-related stress or strain are advisable. As social workers experienced negative work-family spill-over, some effective interventions such as resilience [96] and mindfulness [99] programs would enhance social workers’ stress management, self-care and self-compassion, and, thus, benefit their well-being.

## Figures and Tables

**Table 1:** Descriptive statistics of the Pearl River Delta (PRD) subsample in Chinese Social Work Longitudinal Survey conducted in 2019 (CSWLS 2019).

Cities	DG	FS	GZ	HZ	JM	SZ	ZQ	ZS	ZH	Total *N*
*N* of female (percent of city *N*)	87 (76.99%)	98 (84.67%)	284 (80.45%)	71 (75.53%)	99 (93.42%)	321 (78.29%)	10 (83.33%)	71 (73.20%)	93 (85.32%)	1134 (80.20%)
*M* years of age (*SD*)	28.88 (4.75)	28.84 (5.43)	28.42 (5.75)	30.53 (5.53)	30.07 (6.11)	29.37 (5.23)	29.83 (4.90)	28.96 (5.57)	30.01 (7.19)	29.21 (5.64)
*M* years of education (*SD*)	15.83 (0.76)	15.60 (1.10)	15.46 (1.15)	15.23 (1.36)	15.31 (1.20)	15.63 (1.25)	15.00 (1.04)	15.50 (0.94)	14.84 (1.56)	15.48 (1.21)
*M* years of tenure (*SD*)	4.91 (2.92)	3.53 (2.75)	3.49 (2.58)	3.54 (3.08)	3.92 (2.76)	4.1 (3.03)	2.42 (2.75)	3.75 (2.82)	2.61 (2.97)	3.76 (2.89)
*N* of married (percent of city *N*)	58 (51.33%)	58 (48.33%)	147 (41.64%)	65 (69.15%)	61 (61.32%)	183 (44.63%)	8 (66.67%)	46 (47.42%)	55 (50.46%)	681 (48.16%)
*N* of Assistant Social Worker (percent of city *N*)	34 (30.09%)	16 (13.33%)	45 (12.75%)	16 (17.02%)	18 (16.98%)	80 (19.51%)	1 (8.33%)	15 (15.46%)	6 (5.50%)	231 (16.34%)
*N* of Junior Social Worker (percent of city *N*)	58 (51.33%)	58 (48.33%)	120 (33.99%)	2 (2.13%)	55 (51.89%)	200 (48.78%)	2 (16.67%)	56 (83.58%)	36 (33.03%)	587 (41.51%)
*N*	113	120	353	94	106	410	12	97	109	1414

Note. DG = Dongguan city, FS = Foshan city, GZ = Guangzhou city, HZ = Huizhou city, JM = Jiangmen city, SZ = Shenzhen city, ZQ = Zhaoqing city, ZS = Zhongshan city, ZH = Zhuhai city.

**Table 2:** Zero-order correlational matrix.

	1	2	3	4	5	6	7	8	9	10
1. WS-D	-	0.66 ***	0.56 ***	0.61 ***	−0.23 ***	−0.34 ***	−0.06 *	−0.16 ***	−0.13 ***	0.05
2. WS-M		-	0.68 ***	0.58 ***	−0.30 ***	−0.33 ***	−0.04	−0.16 ***	−0.16 ***	0.08 **
3. WS-S			-	0.55 ***	−0.27 ***	−0.29 ***	−0.08 **	−0.20 ***	−0.18 ***	0.07 **
4. WS-C				-	−0.23 ***	−0.36 ***	−0.07 *	−0.18 ***	−0.16 ***	0.10 ***
5. RA					-	0.43 ***	0.10 ***	0.35 ***	0.26 ***	−0.16 ***
6. RC						-	0.09 **	0.30 ***	0.28 ***	−0.20 ***
7. RO							-	0.15 ***	0.11 ***	−0.09 ***
8. WIF								-	0.54 ***	−0.22 ***
9. FIW									-	−0.20 ***
10. LS										-
Mean	3.72	4.18	4.96	4.1	2.73	2.47	-	1.76	1.33	3.4
SD	1.01	0.82	0.84	0.77	0.62	0.48	-	0.84	0.73	0.77
Cronbach’s *α*	0.89	0.92	0.90	0.90	0.73	0.76	-	0.82	0.83	-

Note. *** *p* < 0.00, ** *p* < 0.01, * *p* < 0.05; WS-D = Work Support from Director; WS-M = Work Support from Manager; WS-S = Work Support from Supervisor; WS-C = Work Support from Coworkers; RA = Role Ambiguity; RC = Role Conflict; RO = Role Overload; WIF = Work-family Interference; FIW = Family-work Interference; LS = Life Satisfaction. RO is a binary variable and the point-biserial correlation was conducted with *r*_pb_ used for a coefficient.

**Table 3:** The standardized SEM results (*n* = 1188).

	->WS	->RA	->RC	->RO	->WIF	->FIW	->LS
Work Support		−0.32 ***	−0.44 ***	−0.04	−0.04	−0.03	−0.01
Role Stress Role Ambiguity					0.25 ***	0.18 ***	−0.00
Role Conflict					0.17 ***	0.17 ***	−0.12 ***
Role Overload					0.09 **	0.05	−0.05
WIF							−0.14 ***
FIW							−0.08 *
Covariates							
Assistant SW	−0.03	−0.03	−0.04	0.14 ***	0.07 *	0.07 *	−0.02
Junior SW	−0.09 *	0.02	−0.04	0.05	−0.03	−0.03	−0.04
SW Job Tenure	−0.15 **	0.03	−0.04	0.04	0.09 *	0.04	0.04
Demographics							
Female	−0.04	−0.05	−0.02	0.02	0.05 *	0.02	0.06 *
Age (years)	0.04	−0.02	−0.04	−0.10 **	0.02	−0.06	−0.04
Married	0.04	−0.07 *	−0.10 **	−0.02	−0.05	−0.02	0.11 **
Education (years)	−0.09 **	0.04	0.06	−0.02	0.04	−0.00	0.04
Factor loadings for indicators of the latent variable Work Support (WS)							
WS ->	Director	Manager	Supervisor	Coworkers			
Factor loading	0.70 ***	0.77 ***	0.74 ***	0.67 ***			
**Model-fit index**							
$\chi^{2}/df$= 2.62; RMSEA = 0.04, with 90% CI = [0.03–0.05]; CFI = 0.98; TLI = 0.94							

Note. *** *p* < 0.00, ** *p* < 0.01, * *p* < 0.05; WS = Work Support; RA= Role Ambiguity; RC = Role Conflict; RO = Role Overload; WIF = Work-family Interference; FIW = Family-work Interference; LS = Life Satisfaction. The covariances of error terms between the WS by coworkers and manager (β=0.18, p<0.00), RA and RC (β=0.36, p<0.00), as well as WIF and FIW (β=0.50, p<0.00) were omitted in the table.
